# Supplementary material for: The Winner Takes it All: Risk Factors and Bayesian Modelling of the Probability of Success in Escaping from Big Cat Predation
Source: Animals (Basel). 2021 Dec 28;12(1):51. doi: 10.3390/ani12010051 (PMC8749502; doi:10.3390/ani12010051)
Supplement: Supplementary file 1 [file animals-12-00051-s001.zip › animals-1429974-supplementary.pdf]

Table S1. Summary of the variables and their respective levels considered during the study of prey escape success in case of attack of big cats.

Cluster	Variable	Type	Levels
Predator	Animal species	Nominal	Lion, leopard, jaguar, tiger, cheetah, puma, snow leopard, panther, serval, ocelot and caracal
	Sex	Nominal	Male/Female
	Age Range	Ordinal	Young/Adult
	Type of attack	Nominal	Single/Combined
	Hunting mode	Nominal	Individual/Partner/Group of more than two animals
	Number of predators	Ordinal	1-30
	Time from first attention to action (seconds)	Numerical	Between 2" and 130".
	Time from first attention to interaction or direct contact with the prey (seconds)	Numerical	Between 4" and 217".
	Predator's point of attachment to its prey	Nominal	Head, neck, back, limb, abdominal region, rear third and wing
	State of the predator at the end of the hunt	Nominal	Healthy, injured and dead
	Hunting Attempts	Ordinal	1 a 7
Prey	Animal species	Nominal	Baboon, Bear, African Stork, Boar, Buffalo, Capibara, Caracal, Catfish, Crocodile, Red deer, Donkey, Duiker, African Elephant, Fennec, Giant Otter, Giraffe, Guanaco, Guinea fowl, Hare, Himalayan Ibex, Hippopotamus, Impala, Jackal, Kudu, Markhor, Rhesus macaque, Pyrenean ibex, Mule deer, Oryx, Ostrich, Owl, Reedbuck, Rhinoceros, Roan antelope, Sloth, Springbok, Steenbok, Topi, Warthog, Wildebeest, Yak, Zebra and Zebu.
	Number of dams	Ordinal	1-2
	Sex	Nominal	Male/Female
	Age Range	Ordinal	Young/Adult
	Social group	Nominal	Alone, in pairs or in a herd
Natural physical environment	Time of day	Nominal	Day/Night
	Atmospheric conditions	Nominal	Sun, clouds, rain, snow and clear night
	Orography	Nominal	Snowy terrain, forest, savannah, jungle, country road, road, rocky terrain, mountain, pond, lake and river
General	Cause of escape	Nominal	Habitat characteristics, group protection, mother's protection of her offspring, relative size of prey, predator's fear of the presence of other potentially threatening element(s), practical experience of the predator, prey defense strategies, relative physical strength of the prey, predator's death during the attack, and prior sighting of the predator by the fleeing prey.
